# A case of pulmonary tuberculosis patient complicated with hemorrhagic fever with renal syndrome and scrub typhus in Yunnan, China: a case report

**DOI:** 10.1186/s12879-023-08416-4

**Published:** 2023-09-27

**Authors:** Hao Huang, Yichen Kong, Hongmin Yin, Zi Yang, Tilian Ren, Yunzhi Zhang

**Affiliations:** 1 Institute of Preventive Medicine, School of Public Health, Dali University, Dali, 67100 Yunnan China; 2 Yunnan Key Laboratory of Screening and Research On Anti-Pathogenic Plant Resources From Western Yunnan, Dali, 67100 Yunnan China; 3 Yunnan University Key Laboratory of Zoonotic Disease Cross-Border Prevention and Quarantine, Dali, 67100 Yunnan China; 4 Xiangyun County People’s Hospital, Xiangyun, 672100 Yunnan China

**Keywords:** Pulmonary tuberculosis, Hemorrhagic fever with renal syndrome, Scrub typhus, *Orthohantavirus*, *Orientia tsutsugamushi*, Co-infection

## Abstract

**Background:**

Hemorrhagic fever with renal syndrome (HFRS) caused by *Orthohantavirus* (OHV) and scrub typhus (ST) caused by *Orientia tsutsugamushi* (OT) are two infectious diseases prevalent in southwest China. Rodents are the natural host and the main source of the two diseases. OT infection to humans is usually resulted from bite of an infective chigger mite on rodents, and OHV is transmitted through contact or inhalation of aerosols and secretions from infected rodent. The use of antibiotics and hormones is crucial for infectious diseases, although the clinical manifestations are not obvious and a definitive diagnosis becomes more difficult in the presence of these drugs. Clinically, fever is the first symptom of these two diseases, and most of them are accompanied by common symptoms such as chills and headaches. The clinical symptoms of these two diseases are very similar and therefore it is not easy to make a differential diagnosis.

**Case presentation:**

In this case, a 44-year-old male famer with pulmonary tuberculosis and a history of working in coal transportation was admitted to the hospital because of respiratory symptoms accompanied by fever, headache, and skin rashes on his body. Biochemical and urinalysis revealed the hepatic and renal injury. The subsequent molecular testing confirmed he suffered from HFRS and scrub typhus simultaneously that the serological and clinical diagnosis could not identify the cause of infection before. Such case has not been reported in Yunnan Province before.

**Conclusion:**

The clinical diagnosis should be combined with serological and nucleic acid testing approaches for differential diagnosis in areas where HFRS and ST are endemic.

**Supplementary Information:**

The online version contains supplementary material available at 10.1186/s12879-023-08416-4.

## Introduction

China has the third highest burden of pulmonary tuberculosis (PTB) in the world [1]. In most cases of PTB, there are few, if any, clinical symptoms or signs in the first contact with Mycobacterium tuberculosis [1]. PTB develops gradually with mild symptoms until the disease progresses to moderate or severe illness. The most common systemic symptom is fever which is low at the beginning, with "night sweats" in some cases [2, 3]. Patients often develop a cough and sputum due to mild hemoptysis with progressive disease. Shortness of breath usually occurs in the advanced stages of the disease due to lesions of the lungs and the lung parenchyma, or some form of tracheobronchial obstruction. As primary pulmonary lesions are usually located below the pleural area, a ruptured pleural cavity can lead to tuberculous pleuritis with pleural effusion and chest pain with typical nonspecific pleuritis symptoms [4].

Scrub typhus (ST), also known as bush typhus or tsutsugamushi disease, is a zoonotic vector-borne disease caused by *Orientia tsutsugamushi* (OT), which is usually transmitted by the bite of a chigger mite and leads to illness from non-specific symptoms, such as fever, headache, swollen lymph nodes, abdominal pain, a rash or scab at the site of the bite, to severe diseases such as bronchitis, pneumonia, myocarditis, and meningitis [5, 6]. The diagnosis of scrub typhus is mainly based on laboratory tests including serological analysis and some molecular assays targeting the 56-kDa type specific antigen (TSA) gene of OT. ST is prevalent predominantly in South and Southeast China, followed by the southwestern regions including the provinces of Yunnan and Sichuan [7, 8]. Karp and Gilliam genotypes of OT are prevalent to the south of the Yangtze River, while Kawasaki and Gilliam genotypes are mainly distributed to the north of the Yangtze River [9, 10]. Especially, Karp, Kato and Gilliam genotypes are the most prevalent ones in Yunnan Province [11].

Hemorrhagic fever with renal syndrome (HFRS), a rodent-borne disease caused by *Orthohantavirus*(OHV), is characterized by fever, renal failure and hemorrhagic symptoms[12]. It is predominantly prevalent in Europe and Asia, and China, accounting for the 90% of all global cases. *Hantaan Orthohantavirus* (HTNV) and *Seoul Orthantavirus* (SEOV) are the two major strains of OHV associated with HFRS in China which are transmitted by the black threaded mouse (*Apodemus agrarius*) and brown house mouse (*Rattus norvegicus*), respectively [13].

Here, we report a rare patient with PTBco-infected by SEOV and Gilliam genotype of OT in Yunnan Province.

## Case presentation

A 44-year-old male patient, residing in a village of Yunnan Province, was admitted to Xiangyun County People's Hospital in March 2021. He had a history of working in coal transportation. He was diagnosed with tuberculosis, tuberculous bronchostenosis, and silicosis by the local disease control center and a hospital due to repeated coughing and phlegm for up to 6 years. When he was discharged from hospital, he accepted the advice of anti-tuberculosis treatment and the anti-tuberculosis HRZE scheme (H: isoniazid, R: rifampicin, Z: pyrazinamide, E: ethambutol). However, after discharge, the patient did not take medicine every day according to the doctor's advice, and still had cough and expectoration symptoms, without obvious regularity of time.After taking anti-tuberculosis drugs on his own for five days, he was admitted to the hospital because of headache and fever for two days with rash, and a high body temperature up to 39.4℃. Rashes were observed on the face, head, neck, chest and abdomen, and limbs, distributing in patches with size ranging from a pinpoint to a grain of rice, which led to pruritus and a red halo at the base without rupture, separated by normal skin. The lips and mouth were slightly cyanotic, and the breath sounds of both lungs were coarse, with scattered moist rales. The other examination results were unremarkable.

By laboratory examination, normal level of blood cells were observed as follows: white blood cells (7.3 × 10^9^/L), hemoglobin (155 g/L), platelets (202 × 10^9^/L), neutrophil percentage (90.6%) and lymphocyte count (7.8 × 10^9^/L). Biochemical tests showed impaired liver function: elevated aspartate aminotransferase(AST) (50U/L), elevated gamma-glutamyl transferase(GGT) (64U/L), slightly lower prealbumin (145.5 mg/L), however normal alanine aminotransferase(ALT) (21U/L). Inflammatory biomarker tests showed procalcitonin at 0.63 ng/mL and C-reactive protein at 59 mg/L. Coagulation analysis showed a prolonged prothrombin time of 13.2 s. Plasma fibrinogen was elevated at 4.80 g/L. Routine urinalysis showed that the patient was positive for urine protein (+ , 0.2 g/L – 1.0 g/L), blood cells (+ , 5 -10 red blood cells observed in 400 × microscopic field), and urinary ketones (+ + , 1.5 mmol/L-3.5 mmol/L), suggesting the presence of impairment of renal function (Supplementary Table 1).

**Fig. 1:**
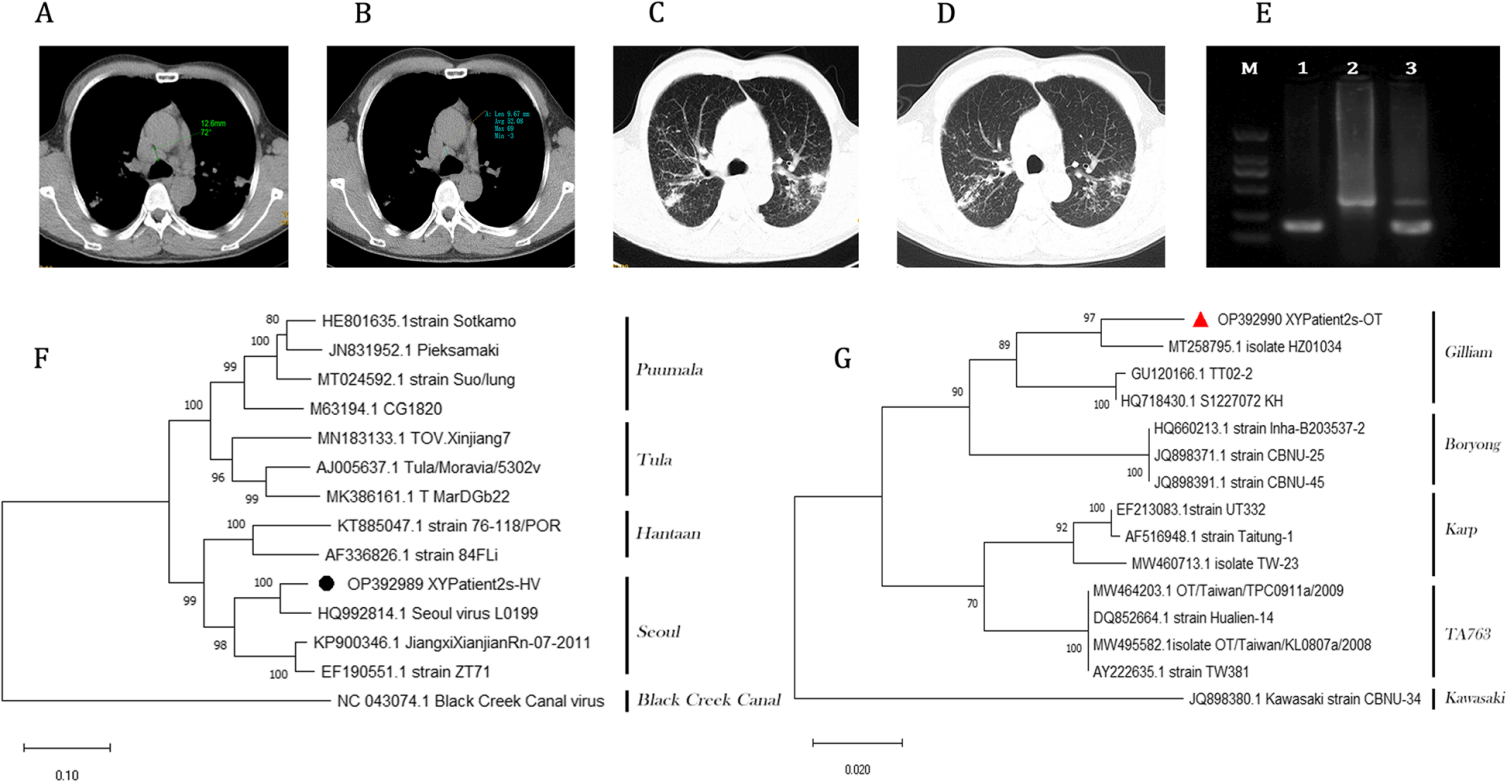
**A** Mediastinal lymph node size measurements on admission. **B** Mediastinal lymph node size measurement map at discharge review. **C** Map of lung lesions on admission. **D** Pulmonary lesion map on discharge review. **E** Gel electrophoresis image of PCR products: M, maker; 1, positive band detected by *Orientia tsutsugamushi*; 2, positive band detected by *Orthohantavirus*; 3, double band of *Orientia tsutsugamushi* and *Orthohantavirus*. **F** Phylogenetic tree of the partial sequence (362 bp) of the L segment of *Orthohantavirus*. Black dot indicates that the patient sample tested in this study. **G** Evolutionary tree of the partial sequence (449 bp) of the 56-kDa type specific antigen gene of *Orientia tsutsugamushi*. Red triangle indicates that the patient sample tested in this study. File XYPatient2s DNA sequenceData.xlsx: DNA sequences of *Orientia tsutsugamushi* and *Seoul orthohantavirus* from XYPatient2S

No acid-fast bacilli were detected by pooling the patient's nocturnal sputum, immediate sputum and morning sputum for testing. According to the patient's chest computed tomography (CT) scan (bilateral lungs and mediastinum) findings, the scattered nodular and lamellar shadows in both lungs were considered as tuberculosis most likely, and the lesions in the lingual segment of the upper lobe of the left lung and the lower lobe of the left lung were probably infectious lesions (Fig. 1A and C). The patient was tested for the antibodies (IgM and IgG) against OHV due to the patient from the natural epidemic foci of HFRS, but the results were negative.

On the first day after admission, the patient suspended his anti- tuberculosis treatment and was given an acetaminophen oral suspension. Subsequently, his temperature decreased and anti-inflammatory treatments were provided with methylprednisolone sodium succinate. The blood gas analysis of the patient showed that the pH value was 7.48, the oxygen partial pressure was 53.2 mmHg, the oxygen saturation was 90.1%, and the potassium ion (K +) was 2.8 mmol/L. The presence of electrolyte disorders (hypokalemia, hyponatremia) was treated with sodium chloride and potassium chloride injections. On the second day after admission, the patient's temperature fluctuated between 38.1–38.7° C, and he still had headache, cough, expectoration and other uncomfortable symptoms. For the symptoms of headache and rash, he took rotundine and ebastine tablets for symptomatic treatment. Later examination showed that the rash on the patient's face, head, chest and neck, and limbs had subsided, and the patient felt relief from the headache. On the third day after admission, the patient continued with symptomatic treatment for anti-infection and cough suppression. The patient sometimes had fever, discomfort, cough and expectoration with little sputum, which was yellow-white sputum. On the Fourth day after admission, the patient was discharged after the rash and fever subsided at the request of the patient and his wife.

In cases where serological and clinical examinations could not identify the specific cause of infection, rash and fever, we performed retrospectively etiological testing of the whole blood and serum from the collected patent previously. TIANamp Virus DNA/RNA extraction kit (DP315, TIANGEN, China) was used to extract viral nucleic acid and DNA of OT which is an intracellular parasitic microorganism with some characteristics of a virus and can be extracted as soon as the OT is lysed and DNA is released from the patient whole blood and serum according to the instructions. Configure the Carrier RNA working solution (final concentration of 1 ug/ul) using Carrier RNA lyophilized powder, buffer GB neutralization, and RNase-Free ddH_2_O according to the reagents provided in the extraction kit. Add 20 μl Proteinase K and 200 μl Carrier RNA working solution to a 200 μl serum or whole blood sample. Close the lid and mix well by vortexing for 15 s. Incubate at 56° C for 15 min, briefly centrifuge, add 250 μl of anhydrous ethanol, and let stand at room temperature for 5 min. Transfer all the mixed solution to the adsorption column, centrifuge at 8000 rpm for 1 min, and discard the filtrate. Add 500 μl buffer GD, centrifuge at 8000 rpm for 1 min, and discard the waste liquid. Add another 600 μl of rinse solution PW, cover the tube cap, let stand for 2 min, centrifuge at 8000 rpm for 1 min, discard the waste liquid, and repeat this step once. Afterwards, 500 μl absolute ethanol was added, centrifuged at 8000 rpm for 1 min, centrifuged at 12,000 rpm for 3 min, discarded the filtrate, opened the lid and left at room temperature for 3 min. Drop 40 μl RNase-Free ddH_2_O in the middle of the adsorption membrane, leave at room temperature for 5 min, centrifuge at 12,000 rpm for 1 min, completely elute the DNA/RNA on the membrane, and store the nucleic acids in a -80 °C freezer.The gene sequence was amplified by one-step nested reverse transcription-polymerase chain reaction(Nested RT-PCR) method using universal primers of OHV according to reference[14]. Nested RT-PCRs were performed using the OneStep RT-PCR kit (Qiagen) for OHV. In each outer reaction, generating an about 600-bp fragment, 2 μl RNA extract was mixed with 10-nmol dNTPs, 2 × reaction buffer, 30-pmol forward primer (HAN-L-F1:5´-ATGTAYGTBAGTGCWGATGC3´), 30-pmol reverse primer (HAN-L-R1: 5´- AACCADTCWGTYCCRTCATC3´), and 1 μl of the supplied.enzyme mix, in a total volume of 25 μl. Cycling conditions were as follows: 30 min at 50℃ and 15 min at 95℃, followed by 35 cycles of 30 s at 94℃, 30 s at 47℃ and 1 min at 72℃. A final elongation step was performed at 72℃ for 10 min. For the inner reaction, generating an about 370-bp fragment, 2 μl of the outer reaction product was used. All reaction and cycling conditions were identical to the ones used for the outer reaction, with the exception of the used primer set (HAN-L-F2: 5´-TGCWGATGCHACIAARTGGTC-3´ and HAN-L-R2: 5´-GCRTCRTCWGARTGRTGDGCAA-3´) and the omission of the reverse transcription step at 50℃.PCR amplification was performed using the 56-kDa TSA gene of OT according to references [15, 16]. The outside primer pair comprised 56KD-F1: 5´-TACATTAGCTGCGGGTATGACA-3´ and 56KD-R1: 5´-CCAGCATAATTCTTCAACCAAG3´. The nested primer pair comprised 56KD-F2: 5´-GAGCAGAGCTAGGTGTTATGTA 3´ and56KD-R2: 5´-TAGGCATTATAGTAGGCTGAGG3´. PCR products were 306 to339 bp for the outside primer pair and 150 to 168 bp for the nested primer pair. PCR conditions were the same for both primer pairs, with initial denaturation for 5 min at 94 °C, followed by 35 cycles of 30 s at 94 °C, 30 s at 50 °C, and 1 min at 72 °C, and a final extension of 5 min at 72 °C.The agarose gel electrophoresis experiment was carried out under the imager (Fig. 1E).The PCR products were purified by gel cutting and sent to a sequencing company (Shanghai Sangon Biotech) for sequencing. The 362 bp sequence of OHV(accession no.OP392989) and the 172 bp DNA sequence of OT were obtained from serum samples. Then primers (ICRA-F2:5 '- CCTCAGTATAATGCCC-3' and ICR8A-R: 5 '- TCCTGCATGACGCTGCAA-3') were designed to obtain 449 bp DNA sequence of tsutsugamushi (accession no.OP392990).

Nucleotide sequence similarity searches in the public databases were assessed by the Basic Local Alignment Search Tool, implemented in the National Center for Biotechnology Information website (www.ncbi.nlm.nih.gov/blast/), using BLASTn, and BLASTn optimized for highly similar sequences (MEGABLAST) and BLASTp, algorithms. In BLAST, it was 92.54% and 97.69% that the highest identity of nucleotide(nt) and amino acid(aa) compared the OHV sequence(OP39298) in this study to SEOV L0199 strain(HQ992814) and SEOV Rn-SHY17 ( ADR32120.1). The highest nt and aa identity compared the obtained OT sequence(OP392990) to *Gilliam* genotype of HZ01034 strain (MT258795.1) and *Orientia tsutsugamushi* str. *Gilliam* (KJV51889.1) was 96.88%, 93.96% respectively (Supplementary Tables 2 and 3).

Phylogenetic trees were analyzed for the obtained OHV sequences (362 bp) and OT sequences (449 bp), and the related sequences retrieved in the Genbank database. The each sequence set was aligned by Clustal-X, and phylogenetic relationships were reconstructed using MEGA X for the initial trees obtained by the maximum likelihood neighbor joining method. In the nucleotide substitution models, the K2 + I and T92 + G models were selected for Bootstrap analysis using 1000 replicates to improve the confidence level of the phylogenetic tree, respectively (Fig. 1F, G).The results showed that the patient had been infected with SEOV of *Orthohantavirus* and Gilliam genotype of *O. tsutsugamushi*.

## Discussion and conclusions

Compared to the CT images of the patient's admission (Fig. 1A-D), the discharge review showed that the patient was effectively treated and recovered in the hospital. Mediastinal lymph nodes in the CT images decreased from 12.6 mm to 9.67 mm after treatment and discharge, which also indicated an inflammatory response in the mediastinal lymph nodes due to the presence of pulmonary tuberculosis and silicosis. The lung lesions seemed to be typical tuberculosis infection. From a large area with blurred boundaries, there was a degree of reduction in the lesion and a clearer peripheral lesion after treatment. If evidence of other infections in the patient was obtained without etiological testing, the patient would have an initial diagnosis of tuberculosis or pulmonary infection.

Since fever is the most common symptom of systemic toxicity in tuberculosis, most of which are low fever for long periods, but the patient in the case sustained high temperature. Combined with the laboratory finding, it cannot be excluded that the patient had fever and rash due to other causes. Patients may experience exacerbations or poor treatment outcomes because they do not follow the principles of early, combined, moderate, regular, and full course of tuberculosis treatment regimens for taking medications. Among many drugs, the most commonly used first-line anti tuberculosis drugs (streptomycin, isoniazid, rifampicin, ethambutol) are clinically effective. The incidence of adverse reactions in conventional doses is low, and liver injury is mainly caused in the form of elevated ALT. The patient did not raise ALT, while AST and GCT were elevated, and there were no other adverse reactions such as gastrointestinal reactions and neurological damage. The patient's past medical history and living habits should not be overlooked in clinical practice, because the ignored factors can have an impact on the development of the disease [17].

Rifampicin is currently used to treat scrub typhus in addition to tuberculosis [18]. Several studies on scrub typhus have also shown that rifampicin which can shorten the time of fever and better control the concurrent inflammation in the patient's lungs is more effective than chloramphenicol [19, 20]. In that case, rifampin, together with anti-tuberculosis drugs, controlled the progression of scrub typhus. Although serology remains the most extensive diagnostic method of Rickettsiosis, molecular methods are complementary to serology. The combination of PCR and serology for acute disease can improve the diagnosis rate and make the diagnosis as clear as possible. The clinical manifestations of rickettsial disease are sometimes atypical and may be similar to other febrile diseases. It is not uncommon for such non-specific symptoms to be misdiagnosed and missed. In many cases, missed proper patients management, physicians may only consider infection of *O. tsutsugamushi* unless the patient has characteristic clinical signs such as eschar [21]. The eschar was not found in this case. The reported case had been infected by Gilliam type of *O. tsutsugamushi* by PCR. Although a kit for the bacterial DNA extraction was not used specifically in this experiment, instead TIANamp Virus DNA/RNA Nucleic acid extraction kit was used to extract DNA from OT, according to the kit instructions, it can be used for DNA extraction from OT.

Studies have shown that early treatment of HFRS with antiviral and hormone treatment attenuates direct viral tissue damage and immunity damage, reduces capillary permeability, suppresses cytokine production and release, and suppresses inflammation [22]. In the case of the patient, the nonspecific manifestation of HFRS was in basic remission after hormonal and antiviral therapy. It was negative that the antibody against *Orthohantavirus*, because the antibody has not been produced or the antibody level has not reached the detectable amount in the early stage of the illness. Therefore, patients with negative serological test results should also be diagnosed in conjunction with other clinical indicators (such as urine volume, platelets, white blood cells, procalcitonin, etc.). And molecular biology testing avoid misdiagnosis and delay [23].

As a seasonal rodent-borne disease, there are certain objective conditions for co-infection of HFRS and scrub typhus, and external environmental factors, including climatic factors, which may play an important role in disease transmission [24]. In this case, *Orthohantavirus* and *O. tsutsugamushi* infection were not initially considered based on the patient's clinical symptoms and serological test results. The case may be considered as a fever of unknown origin or a pulmonary infection. For natural epidemic areas where these two diseases exist at the same time, serological and etiological examinations should be carried out for patients with HFRS or scrub typhus as far as possible to maximize the diagnostic rate, reduce missed cases of co-infection, and provide early treatment to avoid complications caused by delayed treatment or poor treatment results.

### Supplementary Information

**Additional file1: Supplementary Table 1.** The progress, examination and treatment measures of this case. **Supplementary Table 2.** The comparative similarities of partial 56-kDa TSA of Orientia tsutsugamushi at the amino acid and nucleotide levels. **Supplementary Table 3.** BAST results of 449bp of Orientia tsutsugamushi.

**Additional file 2. **DNA sequences of Orientia tsutsugamushi and Seoul orthohantavirus from the reported case in this study.

## Data Availability

All the sequences in this manuscript can be obtained from NCBI database (https://www.ncbi.nlm.nih.gov) via the following accession numbers: XYPatient2S-OT (OP392990); XYPatient2S-HV (OP392989); reference OT sequences (MT258795.1, GU120166.1, HQ718430.1, HQ660213.1, JQ898371.1, JQ898391.1, EF213083.1, AF516948.1, MW460713.1, MW464203.1, DQ852664.1, DQ852664.1, MW495582.1, AY222635.1, JQ898380.1); reference HV sequences (HE801635.1, JN831952.1, MT024592.1, M63194.1, MN183133.1, AJ005637.1, MK386161.1, KT885047.1, AF336826.1, HQ992814.1, KP900346.1, EF190551.1, NC 043074.1).
