# Supplementary material for: A case of pulmonary tuberculosis patient complicated with hemorrhagic fever with renal syndrome and scrub typhus in Yunnan, China: a case report
Source: BMC Infect Dis. 2023 Sep 27;23:631. doi: 10.1186/s12879-023-08416-4 (PMC10523743; doi:10.1186/s12879-023-08416-4)
Supplement: Supplementary file 1 — Additional file1: Supplementary Table 1. The progress, examination and treatment measures of this case. Supplementary Table 2. The comparative similarities of partial 56-kDa TSA of Orientia tsutsugamushi at the amino acid and nucleotide levels. Supplementary Table 3. BAST results of 449bp of Orientia tsutsugamushi. [file 12879_2023_8416_MOESM1_ESM.docx]

**Supplementary Table1. The progress, examination and treatment measures of this case**

| Time | Symptoms | Examination indicators | Treatment measures |
| --- | --- | --- | --- |
| 5 days before admission | Irregular cough and sputum | No information available | Self-administered anti-tuberculosis medication for 5 days, not taking it regularly |
|  |  |  |  |
| 2 days before admission | Chills and fever with headache |  | Self-administered fever-reducing medication, ineffective |
|  |  |  |  |
| The 1^st^ day of admission | Fever, cough, headache aggravation, rash, electrolyte disturbance | T: 39.4℃, PH: 7.48, PO_2_: 53.2mmHg, SaO2c: 90.1%, K+: 2.8mmol/L, Na+: 137mmol/L urine protein (PRO) 1+, blood (BLD) 1+, urine ketone (KET) 2+, prothrombin time delay 13.2s, plasma fibrinogen: 4.81g/L | Vitamin C, Ambroxol, Acetaminophen oral suspension  Methylprednisolone sodium succinate, sodium chloride, potassium chloride |
|  |  |  |  |
|  |  |  |  |
|  |  |  |  |
|  |  |  |  |
|  |  |  |  |
| The 2^nd^ day | Fever, headache, cough, rash subsiding significantly | T: 38.1-38.8℃, chest CT suggests: pulmonary tuberculosis and infectious lesions | Acetaminophen oral suspension, Methylprednisolone sodium succinate, Rotundine, Epastine tablets |
|  |  |  |  |
|  |  |  |  |
|  |  |  |  |
| The 3^rd^ day | Fever, cough, Headache relief at times | T:37.3-37.8℃ | Continue symptomatic treatment such as anti-infection and cough suppression |
|  |  |  |  |
| The 4^th^ day | Fever and rash subsided | T: 37.2-37.5℃ | The patient requested to be discharged with the medication as prescribed by the doctor. |

**Supplementary Table2. The comparative similarities of partial 56-kDa TSA of *Orientia tsutsugamushi* at the amino acid and nucleotide levels**

| Name | 1 | 2 | 3 | 4 | 5 | 6 | 7 |
| --- | --- | --- | --- | --- | --- | --- | --- |
| LANO01000035.1 Gilliam contig.34_1 | - | 95.99 | 65.12 | 69.17 | 96.21 | 85.81 | 96.44 |
| MT258795.1 Gilliam HZ01034 | 94.12 | - | 66.28 | 69.17 | 92.65 | 85.81 | 96.88 |
| MH290214.1 TA763 Asc.MS651.g | 40.76 | 40.76 | - | 95.48 | 63.95 | 64.16 | 65.99 |
| JN587267.1 TA763 BA344_3 | 47.13 | 45.86 | 88.07 | - | 68.06 | 66.67 | 68.33 |
| L04956.1 Boryong | 94.12 | 88.24 | 38.22 | 44.59 | - | 85.13 | 92.65 |
| AY836148.1 Kato Taiwan CDC | 77.78 | 78.43 | 40.76 | 43.95 | 76.47 | - | 87.19 |
| OP392990 XYPatient2S-OT | 94.12 | 94.12 | 40.76 | 44.59 | 88.24 | 80.39 | - |

Note: The upper right is the amino acid similarity. The bottom left shows nucleotide similarity

**Supplementary Table 3. BAST results of 449bp of *Orientia tsutsugamushi***

| Name | Blast n | | | | Blast x | | | |
| --- | --- | --- | --- | --- | --- | --- | --- | --- |
|  | E value | Per.Ident | Accession | Genotype | E value | Per.Ident | Accession | Genotype |
| OT sequence（OP392990） | 0.00 | 96.88% | MT258795.1 | *Gilliam* | 1e-74 | 93.96% | KJV51889.1 | *Gilliam* |
|  | 0.00 | 96.67% | LS398551.1 | *Gilliam* | 2e-69 | 91.28% | UWW11084.1 | *Gilliam* |
|  | 0.00 | 93.54% | AP008981.1 | *Ikeda* | 2e-66 | 89.93% | AAF12762.1 | *Ikeda* |
|  | 0.00 | 93.01% | AM494475.1 | *Boryong* | 2e-64 | 87.25% | AAA26375.1 | *Boryong* |
